# Towards streamlined product information: reporting of transporter-mediated drug interactions

**DOI:** 10.1007/s00228-024-03772-9

**Published:** 2024-11-15

**Authors:** Valeria Asmar, Erik Bergman, Elin Lindhagen, Kim Sherwood, Gabriel Westman, Fabienne Zdenka Gaugaz

**Affiliations:** Swedish Medical Products Agency, Uppsala, Sweden

**Keywords:** Drug-drug interactions, OATP, BCRP, Product information, Standard text, Regulatory science

## Abstract

**Purpose:**

The purpose of this study is to investigate the reporting of risks associated with transporter-mediated drug-drug interactions (DDIs) in medicinal product information and to identify suitable wording for future standardisation of summaries of product characteristics (SmPCs).

**Methods:**

The SmPCs of medicinal products approved in the European Union from 2012 to 2023 were screened for warnings on Organic Anion Transporting Polypeptide 1B1 and 1B3 (OATP1B1 and OATP1B3), and Breast Cancer Resistance Protein (BCRP). An in-house search engine for product information was used. Warnings were categorised into different DDI scenarios based on the SmPC texts.

**Results:**

A total of 192 out of 859 approved medicinal products had SmPC text pertaining to OATP1B1, 1B3 and/or BCRP. The majority of products had text for all three transporters Most texts were located in SmPC Sect. 5.2, followed by Sect. 4.5. Numerous interaction-texts either concluded that the interaction lacked clinical relevance or lacked information on the clinical relevance of the finding. The highest number of SmPC texts indicating a clinically relevant interaction with outlined clinical consequences was found for BCRP. The article also presents SmPC texts for each DDI scenario, which the authors consider as examples of explicit wordings with actionable recommendations.

**Conclusion:**

A potential for improvement of SmPC text for transporter-mediated DDI was identified: Warnings without clinical relevance could be omitted, and some warnings with clinical relevance could be updated to provide actionable recommendations to the prescribers. A selection of unambiguous texts was identified as starting point to generate standard texts.

**Supplementary Information:**

The online version contains supplementary material available at 10.1007/s00228-024-03772-9.

## Introduction

Patients often need to take several medications, which may interact with each other in a pharmacokinetic drug-drug interaction (DDI). DDIs may result in lack of efficacy or increased risk for adverse events. This article focuses on interactions mediated by membrane transporters, which can impact uptake or efflux of drugs from the intestine, liver or kidney. Breast Cancer Resistance Protein (BCRP), Organic Anion Transporting Polypeptide (OATP) 1B1 and 1B3 are transmembrane transporters that are commonly associated with DDIs. Thus, the international transporter consortium (ITC) recommended their investigation during drug product development, along with other transporters [1]. Investigation of the inhibition of these three transporters is a mandatory requirement in a marketing authorisation application [2, 3].

BCRP (gene ABCG2) is an efflux transporter belonging to the ATP-binding cassette transporter family [1]. BCRP is expressed in the gastrointestinal tract, and at the apical membrane of hepatocytes, kidney, brain endothelium, mammary tissue, testis and placenta. BCRP has a broad range of substrates and inhibitors. Examples of sensitive substrates and inhibitors for in vitro and in vivo studies are presented in Table S1 [2].

OATP1B1 and 1B3 are uptake transporters belonging to the SLCO (solute carrier) superfamily [1]. OATP1B1 and 1B3 are primarily expressed in the sinusoidal membrane of hepatocytes. They are involved in transporting anions through membranes while taking up a broad range of substrates such as bile acids and numerous drug substances, including statins.

Concomitant administration of rosuvastatin, an OATP1B1, 1B3 and BCRP substrate, with cyclosporin, an inhibitor of all three transporters, leads to an increased exposure of rosuvastatin (sevenfold higher area under the curve (AUC)) compared to rosuvastatin taken alone. Increased exposure of rosuvastatin has been linked to a risk of skeletal muscle effects such as myositis, myopathy and rhabdomyolysis. Consequently, cyclosporin is contraindicated in patients taking rosuvastatin [4].

In the European Union (EU), new medicinal products can be approved through the centralised procedure which is coordinated by the European Medicines Agency (EMA). The European Commission then formally grants the marketing authorisation in all EU member states [5].

Guidelines have been developed to recommend how to investigate a drug´s interaction potential. The current EU guideline on interactions [3] was last revised in 2012 and came into force in 2013. Prior to this, a guidance had been issued in 1997; however, it did not explicitly recommend the investigation of interactions with drug transporters [6]. Recently, a harmonised guideline across regions, ICH M12, has been finalised and will come into force on November 30th, 2024 [2].

Whenever there are risks associated with DDIs, these should be appropriately warned for in the Summary of Product Characteristics (SmPC) [7]. An SmPC is part of the product information written by the applicant and contains claims for each medicinal product. SmPCs form the basis of information for healthcare professionals on how to use the medicinal product safely and effectively. The structure and the content of the SmPC are defined in guidelines and templates [7, 8]. Once the product is approved, the SmPC and the European Public Assessment Report (EPAR) are published on the EMA website.

Posology adjustments of the drug are presented in SmPC Sect. 4.2, contraindications in Sect. 4.3, special warnings and precautions in Sect. 4.4. The main section pertaining to interactions is Sect. 4.5, where all interactions that impact the use of the drug or its concomitant medication should be described. Interactions with contraceptives are also described in the pregnancy and fertility Sect. 4.6. Further information on interactions can be given in Sect. 5.2, which describes the pharmacokinetic properties of the drug.

Pharmacokinetic DDIs should be investigated following a mechanistic approach and are generally divided in two categories: the effect of the new medicinal product on other drugs and the effect of other drugs on the new medicinal product. These scenarios are also termed perpetrator or precipitant and victim or object, respectively. In vitro investigations are the first step to exclude the risk of clinically relevant interactions. Enzymes and transporters contributing to the main elimination pathways of a drug should be identified. The effects (inhibition or induction) of the drug on common enzymes and transporters should be investigated [2, 3].

Guidelines define which enzymes or transporters should be included in in vitro studies [2, 3, 6]. For transporters, this has evolved from no requirement in the 1997 guidance [6], to the obligation to study transporter inhibition for P glycoprotein (PgP), BCRP, OATP1B1 and 1B3, organic anion transporter OAT1, OAT3 and organic cation transporter OCT2 in the 2012 guidance [3]. In ICH M12, requirements continue to evolve to include additional transporters [2].

For each in vitro assay, a physiologically relevant concentration of the drug is defined depending on the localization of the transporter in the body, and a safety factor is added to account for uncertainties [2, 3]. If no inhibition/induction is observed at the relevant concentration, the risk of DDI can be excluded and constitutes a negative signal. If the risk of DDI cannot be excluded, it is referred to as a positive signal and should be followed up in silico and/or in vivo. For victim interactions, the therapeutic window of the drug must always be considered to determine whether a DDI is clinically relevant or not [2].

The findings are the base for the SmPC texts, which should provide clear guidance for the healthcare professional on how to act upon the DDI risk.

The present retrospective analysis was undertaken to describe how risks and warnings associated with transporter mediated DDIs are expressed in SmPCs. In addition, the study aimed to identify examples of relevant texts for future use in SmPCs to ensure consistent recommendations are given. In particular, the case of a positive in vitro signal without additional follow-up in vivo or in silico data was of interest, as it is challenging to define an adequate level of warnings and risk mitigation when the potential clinical relevance of a positive in vitro screening has not been evaluated. Given their propensity to be involved in DDIs, OATP1B1, 1B3 and BCRP were selected for this analysis. PgP was not included in this analysis, mainly because of the overlap of DDIs with CYP3A4, and the contribution of the transporter to the warning was expected to be challenging to disentangle.

## Methods

### Product information text mining

The data was extracted using an in-house syntactic and semantic search engine for product information cross-search (PICROSS). At the time of search, the PICROSS database was populated with text (sentence-based tokenisation) from SmPC and package leaflet PDF documents from the EMA website with a data lock point on September 7th, 2023 [9, 10]. For this study, only the syntactic search method was used.

### Data analysis

This study focused on medicinal products approved through the central procedure, from 2012, when the EMA interaction guideline [3] was finalised, to the cutoff date of September 7th, 2023.

As of the cutoff date, the product information for 1357 centrally approved medicinal products for human use was searchable in PICROSS. A total of 859 of the 1357 products were initially approved in 2012 or later. PICROSS was searched for “OATP” or “BCRP” in all sections of the SmPC, which generated a list of matching SmPC texts. The result for OATP was then subcategorized into OATP1B1 and 1B3. All SmPCs referred to in this work can be found on the EMA homepage [10]. As SmPCs are updated over time, the texts as of September 7th, 2023 are reproduced in the supporting information excel file.

Inclusion and exclusion criteria were applied as detailed in the supporting information, aiming at retaining one text per active pharmaceutical ingredient (API)—the first approved—and removing possible duplicates.

The texts were classified into different levels of clinical relevance as yes, no or unknown. Whenever several interactions were described in the same SmPC section, the classification reflected the highest level of clinical relevance (i.e. yes > unknown > no). For a text to be considered clinically relevant (category yes), it had to contain an actionable recommendation. In this context, an actionable recommendation could entail a posology adjustment, advice to monitor for adverse events, lack of efficacy or general caution. Texts lacking clinical relevance (category no) were those stipulating that there is no in vitro inhibition or that the API is not a substrate of the transporter, or those confirming the absence of a clinically relevant DDI with an in vivo DDI study.

The category “unknown” covered both texts explicitly stating that the clinical relevance was unknown and texts that could not be classified into the other categories. An example of an unclear text can be found in Lumykras (sotorasib), in Sect. 4.5: “In vitro studies indicate that sotorasib is an inhibitor of human organic anion transporter (OAT)1/3, OATP1B1, Breast Cancer Resistance Protein (BCRP) and P-gp.” [11]. There was no additional text regarding OATP1B1 in the SmPC and the clinical relevance of the DDI was thus classified as “unknown” in the OATP1B1 analysis. For BCRP, additional text was included, which enabled a classification into “yes”, i.e. clinically relevant DDI in the BCRP analysis.

An example of explicit unknown DDI risk is Hepcludex (bulevirtide) [12], where the text in Sect. 4.5 described that OATP inhibition was noted at concentrations relevant for the high dose of buleviritide. This information is followed by precautionary measures to mitigate the DDI risk.

The SmPC texts of products with a potentially clinically relevant interaction (i.e., “yes” or “unknown” according to the categories described above) were further analysed. The consequence of the interaction as described in the text was categorized into the following groups: “contraindication”, “not recommended”, “monitoring”, “caution”, “posology adjustment (dose or timing)” and “no clear consequence outlined”. The category “contraindication” referred to the presence of a warning in SmPC Sect. 4.3 or the use of the word contraindicated in one of the other sections. Similarly, the category “not recommended” was selected whenever that text was present in the warning. The categories “monitoring” and “caution” were differentiated by the type of action that was recommended: Texts recommending monitoring of safety, efficacy or PK were classified in the “monitoring” category, while caution without additional action were classified as such. A posology adjustment could entail a dose reduction during the time of concomitant administration, or a recommendation to stagger the intake of the two drugs. The latter scenario was expected in orally administered products for BCRP, as it is expressed in the gastrointestinal tract.

The consequence of the DDI in a text could appear in several categories. For example, if the text indicated that co-administration was not recommended, but that in case co-administration was needed, monitoring of adverse events and/ or dose adjustments were necessary, all three categories (not recommended, posology adjustment, and monitoring) would be applicable.

The products were also categorised as following based on the text describing DDI data: in vivo, in vitro or both.

The type of interaction was also labelled as “perpetrator” or “victim”, depending on the text content. Positive signals indicated that the risk of clinically relevant interaction could not be excluded. Negative signals were cases where a specific DDI could be excluded. For completeness, DDI scenarios not mentioned in the text were designated as “N/A”, meaning not available. Fixed-dose combination products with one API acting as perpetrator and the other as victim were classified as “other”.

Tables and graphs were created using Excel and GraphPad Prism. Hyperlinks to the SmPC of each product are provided in the supplementary excel document.

## Results

### Description of the dataset

The PICROSS search for “OATP” or “BCRP” resulted in 159, 157 and 177 medicinal products approved between 2012 and 2023 for OATP1B1, OATP1B3 and BCRP, respectively (Figure S1 in the supporting information), representing up to 20.6% of the 859 products approved from 2012 to the cutoff date.

The SmPCs of a total of 192 medicinal products included in the final dataset had text for either OATP1B1 or 1B3 or BCRP. Out of these 192 SmPCs, 143 (74.5%) mentioned all three transporters, while two (1%), three (1.6%) and 29 (15.1%) had warnings for OATP1B1, 1B3 or BCRP only, respectively (Figure S2). There were 10 products (5.2%) that had text for OATP1B1 and 1B3, but not BCRP; four (2.1%) for OATP1B1 and BCRP but not OATP1B3, and one (0.5%) for OATP1B3 and BCRP but not OATP1B1.

For all three studied transporters, 51–62% of the texts were located in SmPC Sect. 5.2 and 32–45% in Sect. 4.5. A few products had texts about BCRP or OATP-interactions in other SmPC sections, including 4.2, 4.3, 4.4, 4.8, 5.1 or 5.3 (Figure S3).

Antineoplastic and immunomodulating agents were the most represented ATC codes (43–46%), followed by anti-infectives for systemic use (18–20%) for all three transporters (Table S2).

The type of data referred to in the text was similar for all three drug transporters (Figure S4). The text reported most often in vitro data only (71–78%), while 15–20% of the SmPCs referred to both in vitro and in vivo data, and 7–9% referred to in vivo data only. The year of approval of drugs included in the dataset is presented in the supplementary information (Table S3 and Figure S5).

### Analysis

BCRP was the transporter with the highest number of texts with clinical relevance (35%), followed by OATP1B1 (16%) and OATP1B3 (14%). The relevance was unknown for 6–14% of texts, while 74, 80 and 51% of texts had no clinical relevance for OATP1B1, 1B3 and BCRP respectively (Figure S6). The clinical relevance of the text is presented separately for each SmPC section in Table 1.

**Table 1:**
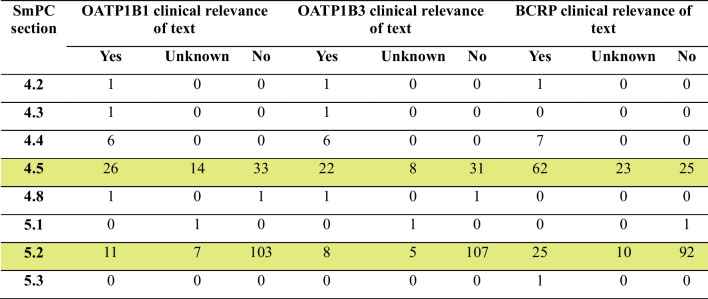
Clinical relevance of the interaction texts stratified by SmPC section

Clinical relevance: whenever several interactions were described in the same SmPC section, the classification reflects the highest clinical relevance (i.e. yes > unknown > no). For the category “yes”, the text had to contain an actionable recommendation. Category “no” included texts stipulating that there is no in vitro inhibition or that the API is not a substrate of the transporter, or texts where the results of an in vivo DDI study were not considered clinically relevant. The category “unknown” covered texts explicitly stating that the clinical relevance was unknown and texts that could not be classified into the other categories. For each transporter, it is possible that the same product can appear in more than one SmPC section

Out of 69 products with texts describing clinically relevant interactions (Figure S7), 15 (22%) products had clinically relevant DDIs for all three transporters. Oxbryta (voxelotor) and Translarna (ataluren) had clinically relevant warnings for OATP1B1 or 1B3 only, respectively, while 41 products (59%) had warnings for BCRP only. There were five products (7%) that had text for OATP1B1 and 1B3, but not BCRP; five products (7%) that had text for OATP1B1 and BCRP but not OATP1B3, and a single product (1.4%, Dovprela—pretomanid) that had text for OATP1B3 and BCRP but not OATP1B1.

Products with a text in Sect. 4.5 with unknown clinical relevance were scrutinized to identify how many were classified as unknown due to inexplicit text or lacked an actionable recommendation (subcategory “missing”). For OATP1B1, it was four “missing” products out of 14 “unknown” products, for OATP1B3 two out of eight and for BCRP four out of 23. These products were Braftovi (encorafenib) for all three transporters, Vemlidy (TFA) for OATPs, Xtandi (enzalutamide) and Lumykras (sotorasib) for OATP1B1, Jardiance (empagliflozin), Uptravi (selexipag) and Retsevmo (selpercatinib) for BCRP.

The type of data (in vitro or in vivo) described in the text was assessed stratified by clinical relevance (Table S4). The majority of DDI text without clinical relevance was based on in vitro data only for all three transporters. In the category “unknown”, in vitro data was also predominant, while texts about clinically relevant DDIs relied more often on in vivo data. The majority of the clinically relevant signals were accompanied by in vivo data. For positive perpetrator signals, 10 out of 20 clinically relevant OATP1B1 texts were supported by in vivo data. Four texts indicated clinically relevant OATP1B1 victim DDI and all were supported by in vivo data. The numbers were similar for OATP1B3 (Table S4). Twenty-three out of 26 clinically relevant BCRP perpetrator texts were supported by in vivo data, while the corresponding number for clinically relevant BCRP victim texts was 19 out of 30.

The type of interaction reported in the SmPC text was categorised into “perpetrator”, “victim” or “other” (in fixed dose combinations that interacted with each other) and subcategorised into text indicating the presence or the absence of an interaction risk. The texts reflecting the lack of clinically relevant interaction as either perpetrator and/or victim amounted to up to 68% for OATPs, and 33% for BCRP (Figure S8 and Table S5).

**Fig. 1:**
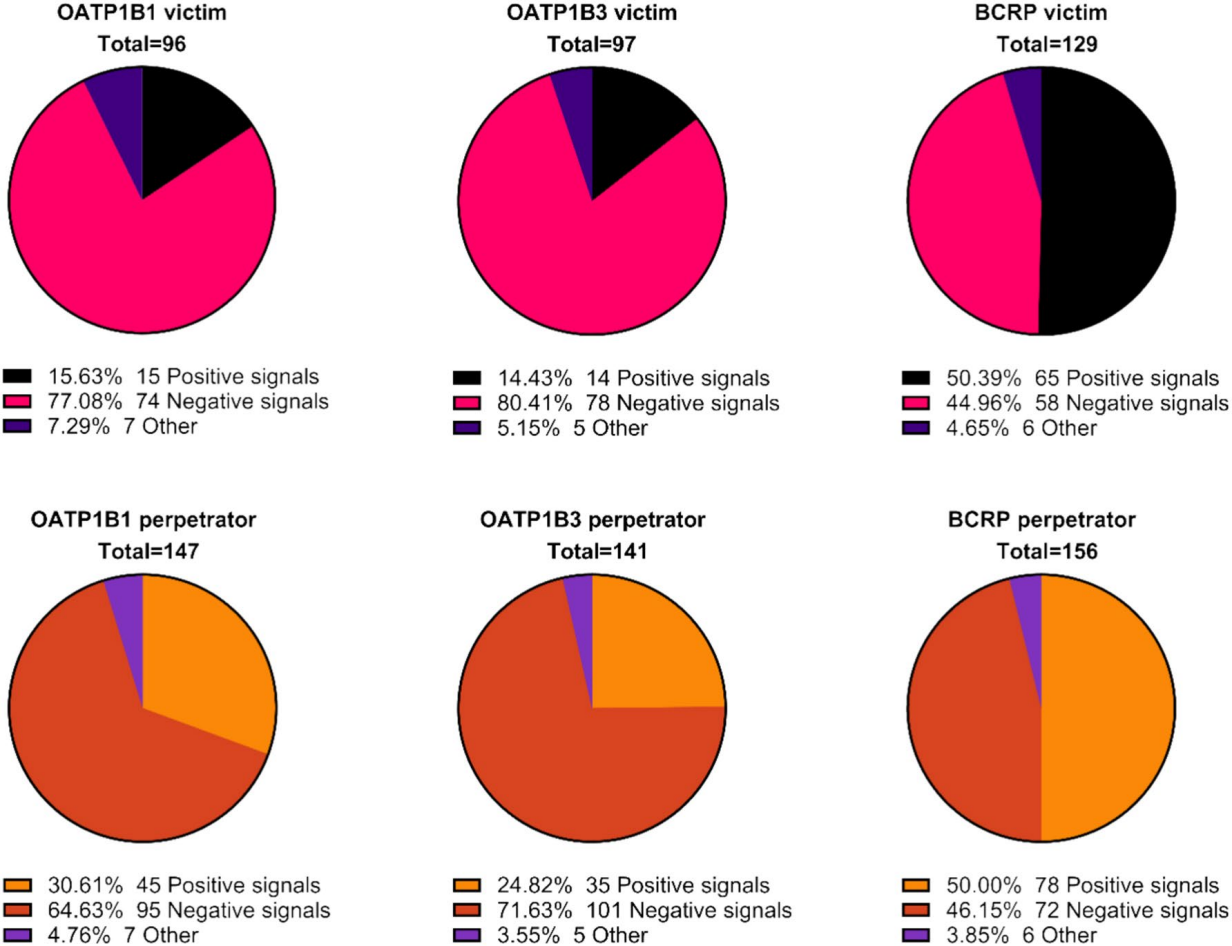
Described interactions (all SmPC sections) categorised into “perpetrator”, “victim” or “other” for OATP1B1, 1B3 and BCRP. Maximum one signal per DDI type and transporter. Other refers to fixed dose combination products where APIs interact with each other

Considering the products that had text on OATP victim aspects, up to 80% of these described a negative signal. For BCRP victim aspects, 45% described a negative signal, while 50% described a positive DDI signal (Fig. 1 top panel).

The number of texts describing perpetrator interactions was higher. 25–50% described a positive perpetrator signal, with 50% for BCRP. In contrast, up to 72% described negative signals (Fig. 1 bottom panel).

For OATP1B1, 25% of the products had a warning that they were perpetrators, while 6% were victim, and 3% were both. The products that were both perpetrator and victim were: Entresto (sacubitril and valsartan), Prevymis (letermovir), Veklury (remdesivir), Evrenzo (Roxadustat) and Aquipta (atogepant). For OATP1B3, 20% were perpetrator, 6% victim and 3% were both (same products as OATP1B1, except Evrenzo). For BCRP, 27% were perpetrator, 20% victim and 17% (*n* = 30) were both (Figure S8 and Table S5).

The consequence of the text for each DDI was examined. There were only few texts informing on a contraindication, up to three per transporter and type of interaction (Table 2). The three products contraindicated due to perpetrator DDI were the same across all three transporters. These products were Harvoni (ledipasvir, sofosbuvir), Maviret (glecaprevir, pibrentasvir) and Vosevi (sofosbuvir, velpatasvir, voxilaprevir). The products contraindicated due to victim DDI were Zepatier (elbasvir, gazoprevir), Maviret and Vosevi: only Maviret was contraindicated for BCRP, while all three products were contraindicated for both OATPs. All four products that had contraindications are indicated for the treatment of hepatitis C virus infection.

**Table 2:** Described consequences of potentially clinically relevant interaction

Described interaction	OATP1B1 perpetrator	OATP1B1 victim	OATP1B3 perpetrator	OATP1B3 victim	BCRP perpetrator	BCRP victim
Contraindication	3	3	3	3	3	1
Monitoring	12	0	11	0	28	7
Posology adjustment (dose or timing)	7	2	5	2	19	6
Not recommended	5	2	5	2	7	4
Caution	13	4	10	4	20	7
No clear consequence outlined	12	3	6	2	21	14
Number of products with potentially clinically relevant DDI^a^	42	42	31	31	72	24

^a^Independent of the type of DDI (perpetrator/victim). The consequence of the DDI in a text could appear in multiple categories (see “Methods”)

Up to 19 texts recommended a posology adjustment in dose or timing (Table 2). The number of products recommending caution was up to 20, and 28 for monitoring. No clear consequence of the interaction was outlined in up to 21 products.

### Towards standard texts

In an effort to promote regulatory consistency and as a starting point for the development of standard texts, unambiguous SmPC texts were identified for some common scenarios. Table 3 presents products with SmPC text that was considered explicit, and the full SmPC text for the respective interaction is presented for perpetrator and victim interactions in Table S6 and S7, respectively.

**Table 3:** Examples of unambiguous SmPC texts for different DDI scenarios

Scenario	Perpetrator	Victim
Contraindication of concomitant use	No example^a^	OATPs: Zepatier (elbasvir, gazoprevir) [13]
Concomitant use not recommended or only with monitoring	OATPs and BCRP: Nubeqa (darolutamide) [14]	BCRP: Venclyxto (venetoclax) (but also perpetrator) [13]
Monitoring recommended including dose adjustment	OATPs and BCRP: Rukobia (fostemsavir) [15]	OATPs: Aquipta (atogepant) [16] BCRP: Adempas (riociguat) [17]
Concomitant use not recommended and dose staggering	BCRP: Fotivda (tivozanib) [18], Jakavi (ruxolitinib) [19], Mekinist (trametinib) [20], Imbruvica (ibrutinib) [21]	BCRP: Giotrif (afatinib) [22]
Precautions when no in vivo data are available	OATP1B1 and BCRP: Rozlytrek (entrectinib) [23] OATP1B3 and BCRP: Dovprela (pretomanid) [24]	BCRP: Talzenna (talazoparib) [25], Rubraca (rucaparib) [26], Stivarga (regorafenib) [27], Mavenclad (cladribine) [28]
In vitro data with no clinical relevance^b^	OATPs: Iclusig (ponatinib) [29]^b^	OATPs: Iclusig (ponatinib) [29]^b^

Selected texts can be found in the respective SmPCs in Sects. 4.2, 4.3, 4.4, 4.5 and 5.2

^a^Perpetrator DDIs resulting in contraindications in this analysis were for anti-infectives and presented in table format, which is not a preferred format for products in other classes. There is thus no example proposed

^b^Text for SmPC Sect. 5.2 only. The authors propose to omit this information in SmPCs due to the lack of clinical relevance

Rozlytrek (entrectinib) and Talzenna (talazoparib) were two of the selected products with explicit texts on perpetrator and victim aspects, respectively, to inform on precautions to be taken when a DDI risk was identified in vitro but no in vivo data is available. The text referred to is reproduced here:

*Rozlytrek: 4.5: Inhibition of BCRP was observed in in vitro studies. The clinical relevance of this inhibition is unknown, but caution is advised when sensitive oral BCRP substrates (e.g. methotrexate, mitoxantrone, topotecan, lapatinib) are co-administered with entrectinib, due to the risk of increased absorption. […] In vitro data indicate that entrectinib has weak inhibitory potential towards organic anion-transporting polypeptide (OATP)1B1. The clinical relevance of this inhibition is unknown, but caution is advised when sensitive oral OATP1B1 substrates (e.g. atorvastatin, pravastatin, rosuvastatin repaglinide, bosentan) are co-administered with entrectinib, due to the risk of increased absorption* [11].

*Talzenna: 4.5: Talazoparib is a substrate for drug transporters P-gp and Breast Cancer Resistance Protein (BCRP) and it is mainly eliminated by renal clearance as unchanged compound. […] The effect of BCRP inhibitors on PK of talazoparib has not been studied in vivo. Co-administration of talazoparib with BCRP inhibitors may increase talazoparib exposure. Concomitant use of strong BCRP inhibitors (including but not limited to curcumin and cyclosporine) should be avoided. If co-administration of strong BCRP inhibitors cannot be avoided, patient should be monitored for potential increased adverse reactions.*

*5.2: Talazoparib is a substrate of P-gp and BCRP transporters* [25].

## Discussion

Similar studies to the present one have been conducted in the past, albeit focussing on different aspects. Both Yu et al. and Maeda et al. reviewed PK interactions broadly, including CYP mediated DDIs and focussed on the effect size of DDIs for drugs approved by the FDA and the Japanese authority PMDA, respectively [30, 31]. No new strong inhibitors of transporters were identified among drugs approved by the FDA in 2020 [30]. Agarwal and colleagues [32] identified an increase in information about transporters in FDA-approved package inserts before and after the introduction of the first guidelines mentioning transporters. The predominant transporter in their analysis was PgP, but there were a few texts about OATPs and BCRP. Recommendations were provided on how to present this information in FDA package inserts. This analysis was followed up by Fan et al. who investigated the transporter-related post-marketing requirements by the FDA from 1999 to 2015 and identified that 65% led to updates in labelling once fulfilled [33].

The present analysis brings in new perspectives on the EU product information. The present analysis focussed on the message conveyed by the SmPC text, not on the underlying data and their interpretation. A prerequisite was that the transporter was mentioned in the SmPC. The absence of hit could be due to the DDI risk being excluded or considered to lack clinical relevance but could also be due to a spelling error in the SmPC. The DDI could also be described without specification of its mechanism, for example when mentioning a specific substance or substance class such as HMG-CoA reductase inhibitors [4, 34].

The dataset showed a large degree of similarity between OATP1B1 and 1B3 texts throughout the analysis. This was anticipated given that the transporters belong to the same family. Substances are often substrates of both. Inhibition of both transporters is commonly seen, albeit sometimes at different concentrations, which may impact the clinical relevance of the interaction, for example in Lynparza (Olaparib) [35].

The BCRP text dataset shared some similarities with the OATPs; however, the warnings tended to be of higher clinical relevance for both perpetrator and victim interactions compared to OATP texts. The higher number of signals for perpetrator interactions could be due to higher in vitro concentrations being tested for orally administered drugs for BCRP. The higher concentrations are needed to cover for the risk of DDI in the gastrointestinal tract, which is not applicable to OATP1B1 or 1B3. BCRP substrates and inhibitors also overlap with PgP [36, 37]. Consequently, it is not always possible to select an in vivo study design that would enable the isolation of a single transporter effect. Further overlaps are also known with enzymes, where the enzyme effect may be larger and the main responsible for the interaction, such as for riociguat (Adempas), where DDIs with multipathway inhibitors of CYP, PgP and BCRP are warned for [17].

Interactions between statins and OATP inhibitors are well-known due to the established link to myopathy and in extreme cases rhabdomyolysis [38, 39]. Rosuvastatin, one of the probe substrates recommended for in vivo interaction studies, is a substrate of all three studied transporters [4]. Thus, many perpetrator warning texts refer to the same substance group as potential victims. It must be noted that the statins themselves were not part of this dataset as they were approved prior to 2012. The widespread use of statins [40] could also have led to an increased interest for including information about transporter DDIs.

Some major areas of possible SmPC improvement were identified. The analysis revealed that the majority of texts about OATPs, and a large number of texts about BCRP indicated the absence of an interaction (Table 1, Fig. 1), which is not in line with the SmPC guideline [7]. The location of these texts describing the lack of a clinically relevant DDI was astonishing, as many were described in Sect. 4.5. While not factually incorrect, this information may distract the prescriber from more relevant information. It is thus recommended to omit such text. The reader should be able to rely on that the absence of a DDI risk description indicates that data on all transporters covered in the current DDI guideline was available and a DDI risk was excluded. However, there are exceptions where results of an in vivo study denoting the lack of DDI could still be presented in Sect. 4.5, for example in therapeutic areas known for the propensity of DDIs (antivirals) [7]. According to the guideline, information on the involvement of transporters in the absorption or elimination of a drug could be presented in Sect. 5.2. However, there is no recommendation to inform on the lack of inhibition (perpetrator data) in Sect. 5.2. Despite this, 12% (for OATP1B3) of all approved products during the period included negative transporter data in 5.2 and could thus benefit from a SmPC revision to remove irrelevant information. It should be noted that the policy regarding the presentation of interaction data differs between the EMA and FDA. The EMA considers that information of clinical relevance should be described, whereas the FDA encourages the presentation of data denoting the lack of interaction in the clinical pharmacology section [41–43].

In addition, many texts were unclear with respect to whether an action was anticipated from the prescriber. For products of the category “unknown” which were classified as such due to the lack of explicit recommendation, additional information may be considered. Actionable recommendations could be added to the SmPC to support the healthcare professionals with information on how to use the medicine safely and effectively.

The case of a positive in vitro signal without additional in vivo or in silico data was of interest, as it is challenging to define an adequate level of warning and risk mitigation. In the OATP1B1 perpetrator analysis, 10 DDIs were classified as clinically relevant, and 11 as of “unknown” clinical relevance when based only on in vitro data. For OATP1B3, eight DDIs were classified as clinically relevant, and four as “unknown”, while for BCRP 26 DDIs were classified as clinically relevant, and 13 as of “unknown”. There was thus a high frequency of observed in vitro interactions without clearly defined regulatory or clinical action to follow (i.e. request for an in vivo study or for a warning text). The SmPC texts addressing this scenario varied from lack of warning to different levels of caution, all based on an IC50 value compared with a reference therapeutic concentration. Even if the in vitro experiments only provide a “yes”/ “no” answer to the question whether a clinically relevant interaction can be excluded, sometimes the applicant and the assessor also consider how close the IC50 is to the relevant cutoff. For victim interactions, it could be possible to identify the clinical relevance if information on the therapeutic window is available.

Defining the level of clinical relevance was the most challenging and subjective part of this project. Available DDI-checking tools were consulted to support the classification of clinical relevance or action [44, 45]. As there was no consensus on how to classify the DDIs, the analysis was data driven. The consequence categories (Table 2) differ from the scenarios selected as potential starting points for standard texts (Table 3), as the latter were based on the experience gathered when assessing new drug applications.

Some texts were classified as of “unknown” or “lacking” clinical relevance despite a positive signal. These could be cases such as Mulpleo (lusutrombopab) [46] where the clinical DDI study demonstrated an impact on PK, but the therapeutic window was sufficiently broad to conclude on the lack of clinical relevance. In other cases, there were in vivo data but the relevance of the DDI was uncertain, such as for Orgovyx (relugolix) [47].

All contraindications identified in this analysis were found in hepatitis C products [13, 48–50]. The clinically more serious DDIs, i.e. contraindications, were generally unambiguous. DDIs slightly less severe, where dose adjustments or monitoring may suffice, had a high degree of variability in the provided [not always] actionable recommendation. This highlights the larger room for interpretation of SmPC information and that standard texts would be helpful.

Whenever a DDI is potentially clinically relevant, it is vital that this message is clearly conveyed to the prescriber with a description of any required action to take. This is important as warning texts can be taken out of context and misinterpreted. An effective text would include information regarding what to monitor, and the consequences of a higher or lower exposure, along with relevant examples of substances. Keeping the SmPC updated with relevant DDI examples is challenging over time. At the EMA level, there is no listing of clinically relevant substrates and inhibitors of the different transporters. The clinical relevance may also differ depending on the therapeutic context, which could be the reason why examples given for the same mechanistic DDI may vary. The FDA however has published a list that may be used as a starting point to find relevant examples of concomitant drugs at risk [51].

The next step of the project was to identify SmPC texts that were perceived as easy to understand and actionable, as a basis for developing standard texts for different DDI scenarios (Tables 3, S6 and S7). There are ongoing projects aiming at the introduction of standard sentences for the improvement of package leaflet [52]. Other initiatives analysed the overall content in product information to understand where harmonisation would have the greatest impact [9].

Standard texts would be particularly helpful in the perpetrator scenario. While there are currently no standard texts to describe DDIs with transporters, the scenarios identified here (Tables 3, S6 and S7) and the corresponding example SmPC texts could form the basis for developing such standard texts. These could be used not only for OATPs and BCRP but could also be adapted to DDIs with other transporters and enzymes.

Harmonised texts for DDIs would result in streamlined, unambiguous texts where the SmPC reader would not be left to make their own interpretation. Besides achieving consistency for similar scenarios, the DDI texts could be harmonised across indications. Further advantages could be that the availability of multiple choices of standardised texts for each DDI scenario could simplify the writing of SmPCs for applicants and reduce assessment time for regulators. By using reference data vocabularies on standard sentences as structured data, spelling errors would also be avoided. Standardised texts are compatible with EMAs electronic product information initiative [53]. Other projects also aim at developing standard texts, for example for the reproduction and lactation sections of the product information [54]. It must be noted that not all scenarios may be covered by standard sentences, and it will remain essential that the documents are created and assessed by experts, keeping the human in the loop throughout the process. Ultimately, generating standard texts for DDIs with the possibility to technically structure information for better filtering and searchability would be most valuable for healthcare professionals in facilitating interoperability with e-health systems using standardised information. This would ensure potential DDIs are properly warned for and handled adequately, maintaining efficacy and safety for each patient regardless of concomitant medications.

## Supplementary Information

Below is the link to the electronic supplementary material.

Supplementary file1 (DOCX 955 KB)

Supplementary file2 (XLSX 1.18 MB)

## Data Availability

Data is provided within the manuscript and supplementary information files.
