# Supplementary material for: Towards streamlined product information: reporting of transporter-mediated drug interactions
Source: Eur J Clin Pharmacol. 2024 Nov 15;81(1):151–61. doi: 10.1007/s00228-024-03772-9 (PMC11695577; doi:10.1007/s00228-024-03772-9)
Supplement: Supplementary file 1 — Supplementary file1 (DOCX 955 KB) [file 228_2024_3772_MOESM1_ESM.docx]

**Title:** Towards streamlined product information: Reporting of transporter-mediated drug interactions

Valeria Asmar

Erik Bergman

Elin Lindhagen

Kim Sherwood

Gabriel Westman <https://orcid.org/0000-0001-9402-772X>

Fabienne Zdenka Gaugaz <https://orcid.org/0000-0001-9171-8891>

Swedish Medical Products Agency, Uppsala, Sweden

**Corresponding author:**

Fabienne Z. Gaugaz

Swedish Medical Products Agency, Uppsala, Sweden

[fabienne.gaugaz@lakemedelsverket.se](mailto:fabienne.gaugaz@lakemedelsverket.se)

**Supporting information**

**Methods**

Inclusion criteria:

1. Medicinal products for human use approved through EMA’s central procedure containing “OATP” or “BCRP” in their SmPC text in sections 1 through 6, as retrieved from a search in PICROSS.

2. Medicinal products with an initial approval date by the European Commission between 2012 and September 7^th^, 2023, as stated in SmPC section 9 (date of first authorisation).

Exclusion criteria:

1. Medicinal products with the same API containing DDI text identical to another medicinal product approved prior to 2012. This category refers to products such as: generics, duplicates, fixed dose combinations. An example of excluded products is Zytiga (abiraterone), approved in 2011 and its generics Abiraterone Accord and Abiraterone Mylan approved in 2021.

2. Medicinal products with the same API containing DDI text identical to another medicinal product approved between 2012-23. In these cases, only one of the products was retained (i.e., the oldest). This category refers to products such as: generics, duplicates, fixed dose combinations, medicinal products approved under several names for different indications. Such an example is Ofev and Vargatef, which both contain nintedanib but have different indications and the same DDI text [1, 2]. Vargatef was retained in the dataset, as it was approved in 2014, a few months before Ofev, which was excluded from the dataset. Another example is Vemlidy (tenofovir alafenamide (TFA) [3]), approved in 2017), which was retained in the dataset, while products approved later containing TFA as part of a fixed dose combination and had the same warning text for TFA were excluded (Symtuza, Genvoya, Odefsey, and Descovy).

3. For fixed-dose combination products containing one or more old APIs (i.e., API approved in another product before 2012) as well as novel API, the product was included, but only the novel API text was analysed. Example of such scenario is Viekirax [4], an antiviral drug approved in 2015, and contains the following three APIs: ombitasvir, paritaprevir and ritonavir. Viekirax was included in the analysis, but the analysis only considered the APIs approved post-2012 (i.e., ombitasvir and paritaprevir), while ritonavir was excluded from the analysis. Ritonavir, marketed as Norvir [5] was approved in 1996.

4. For OATP: text pertaining another transporter from the OATP family, OATP2B1 or 1A2 for example.

**Results**

**Figure S1** Flow-chart illustrating the final number of products included in the analysis for each transporter after the application of inclusion and exclusion criteria.

n=4

n=143

n=10

n=29

n=3

n=2

n=1

**Figure S2** Venn diagram of identified SmPC texts for OATP1B1, 1B3 and BCRP after applying the study inclusion and exclusion criteria


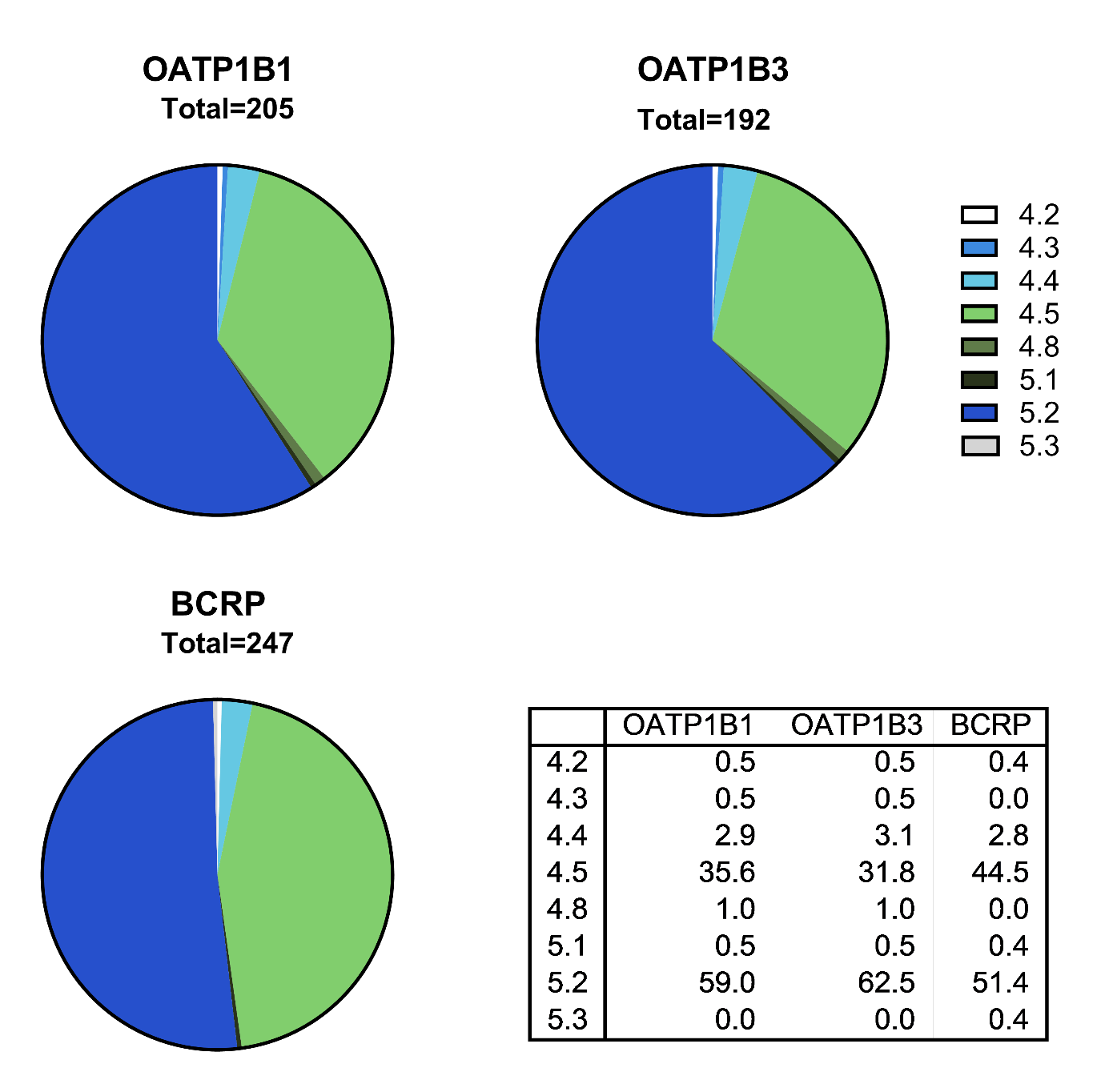
Figure S3 Circle diagram and summary (%) of reporting of interaction texts in SmPC sections for OATP1B1, 1B3 and BCRP.


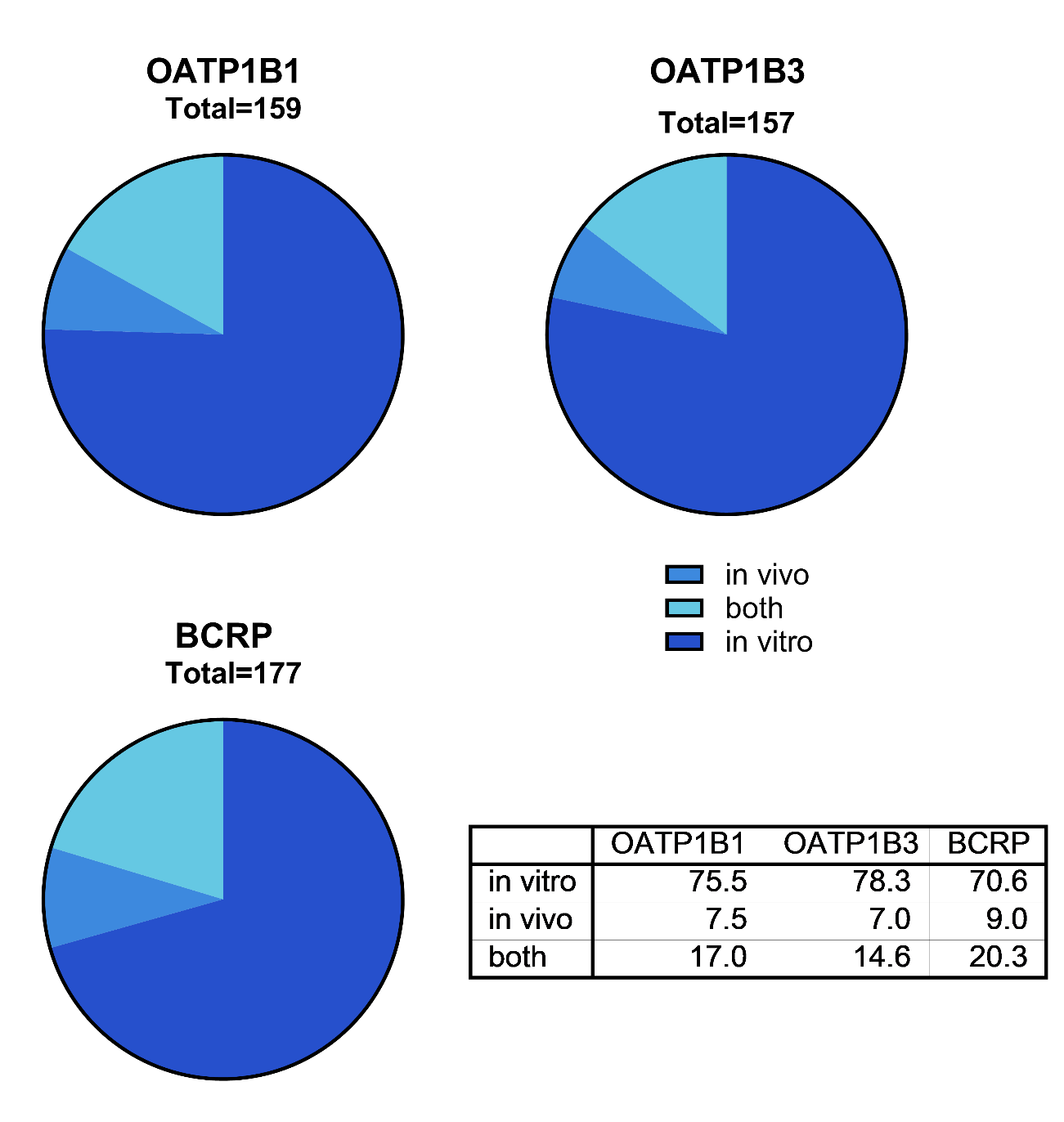


Figure S4 Circle diagram and summary (%) of the type of data (in vitro/in vivo/both) described in the SmPC texts about OATP1B1, 1B3 and BCRP interactions


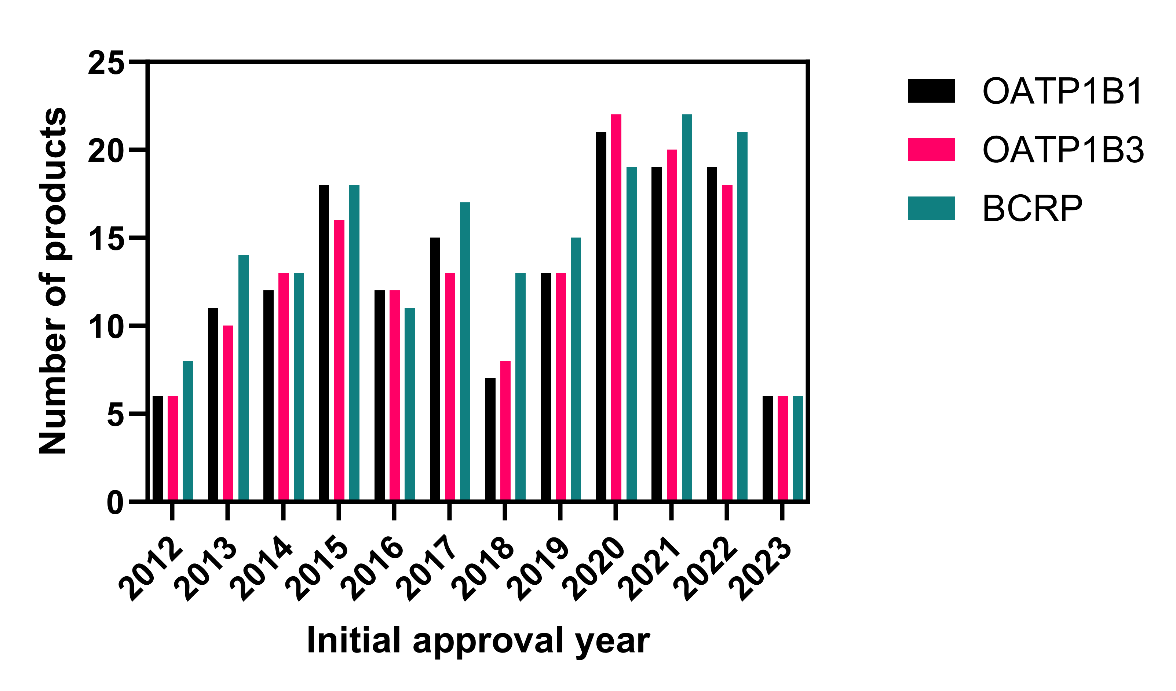


**Figure S5** Number of approved products with SmPC text on OATP1B1, 1B3 or BCRP per approval year


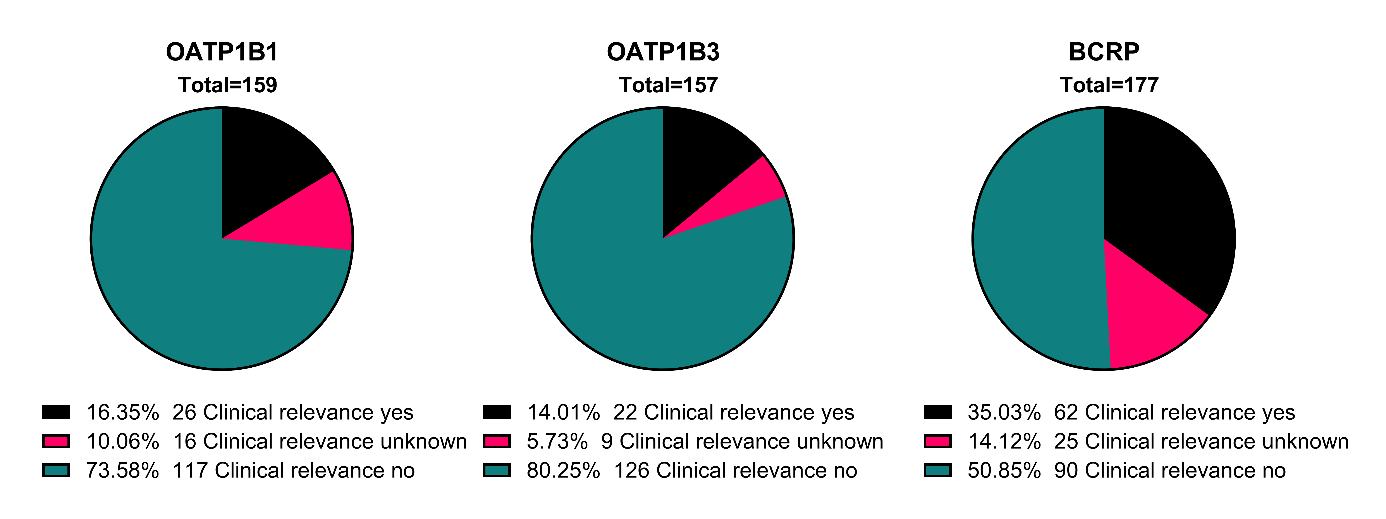


**Figure S6** Clinical relevance of SmPC text on OATP1B1, 1B3 or BCRP. Clinical relevance: whenever several interactions were described for a product, the classification reflects the highest clinical relevance (ie yes > unknown > no). For category “yes”, the text had to contain an actionable recommendation. Category “no” included texts stipulating that there is no in vitro inhibition or that the API is not a substrate of the transporter, or those with in vivo results were the DDI was not considered clinically relevant. The category “unknown” coveredtexts explicitly stating that relevance was unknown and texts that could not be classified into the other categories.

n=5

n=15

n=5

n=41

n=1

n=1

n=1

**Figure S7** Venn diagram of products with SmPC texts describing clinically relevant interactions for OATP1B1, 1B3 and BCRP (relevance category “yes”). Of 69 products with texts describing clinically relevant interactions, the 15 (22%) following products had clinically relevant DDIs for all three transporters: Aubagio (teriflunomide), Gavreto (pralsetinib), Harvoni (ledipasvir, sofosbuvir), Kaftrio (ivacaftor, tezacaftor, elexacaftor), Kisqali (ribociclib), Lorviqua (lorlatinib), Maviret (glecaprevir, pibrentasvir), Nubeqa (darolutamide), Prevymis (letermovir), Rukobia (fostemsavir), Tybost (cobicistat), Viekirax (ombitasvir, paritaprevir, ritonavir), Vosevi (sofosbuvir, velpatasvir, voxilaprevir), Xenleta (Lefamulin) and Zepatier (elbasvir, gazoprevir). Of the 69 products, only Oxbryta (voxelotor) and Translarna (ataluren) had clinically relevant warnings for OATP1B1 or 1B3 only, respectively, while 41 products (59%) had warnings for BCRP only. There were 5 products (7%) that had text for OATP1B1 and 1B3, but not BCRP; 5 (7%) for OATP1B1 and BCRP but not OATP1B3, and one (1.4%, Dovprela - pretomanid) for OATP1B3 and BCRP but not OATP1B1.


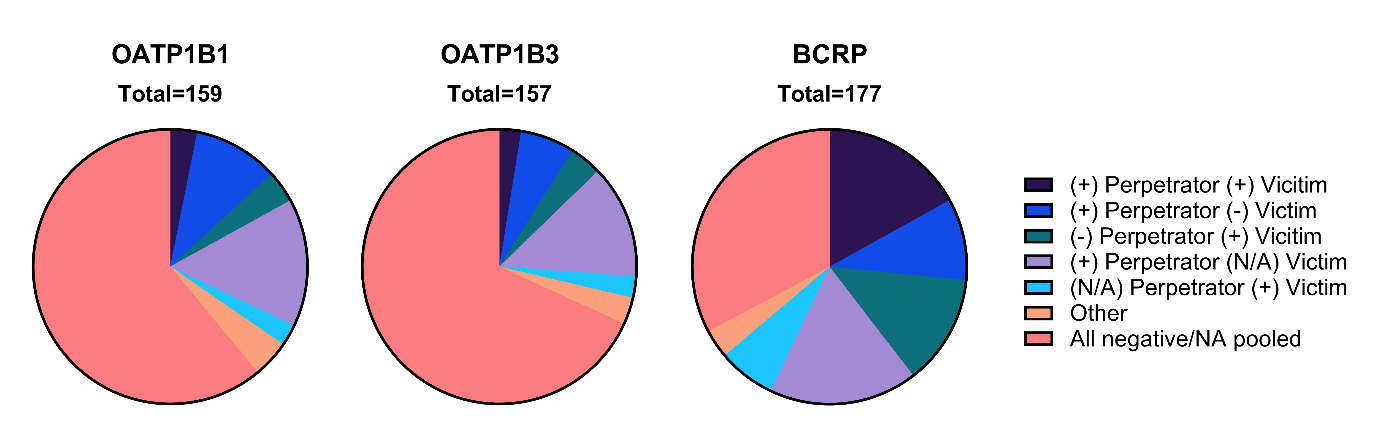


**Figure S8** OATP1B1, 1B3 or BCRP interaction signals as described in the SmPC, stratified by type of interaction as “perpetrator” or “victim”. The presence of a signal is denoted by + (positive signal), a negative signal by minus and if only one aspect (victim or perpetrator) is addressed in the text, the “other” is noted as not available (N/A).

**Table S1** recommended substrates and inhibitors in DDI assessment, according to ICH M12 [6]

| Transporter | OATP1B1, 1B3 | BCRP |
| --- | --- | --- |
| In vitro substrate | Cholecystokinin octapeptide (CCK-8, selective for OATP1B3),  Estradiol-17β-glucuronide,  Pitavastatin,  Pravastatin,  Rosuvastatin | Estrone-3-sulfate,  2-amino-1-methyl-6-phenylimidazo[4,5-b]pyridine (PhIP),  Prazosin,  Rosuvastatin,  Sulfasalazine |
| In vitro inhibitor | Bromosulfophthalein (BSP),  Cyclosporine,  Rifampin,  Rifamycin SV | Fumitremorgin C,  GF120918 (dual P-gp/BCRP inhibitor),  Ko143,  Novobiocin |
| In vivo substrate | Atorvastatin (also a substrate of BCRP, P-gp, and CYP3A)  Bosentan  Pitavastatin  Pravastatin (also a substrate of MRP2 and OAT3)  Rosuvastatin (also a substrate for BCRP, OAT3, and OATP2B1)  Simvastatin acid (also a substrate of CYP3A) | Rosuvastatin (also a substrate for OATP1B1, 1B3, 2B1, and OAT3)  Sulfasalazine (only affected by intestinal BCRP) |
| In vivo inhibitor | Rifampin (single dose, also inhibits P-gp)  Cyclosporine (also inhibits CYP3A, MRP2, P-gp and BCRP) | Cyclosporine (also inhibits CYP3A, MRP2, OATP1B1, OATP1B1B3, and P-gp)  Darolutamide  Fostamatinib (also inhibits P-gp) |

**Table S2** Classification of the dataset into therapeutic areas based on the ATC code.

| ATC code first letter | Therapeutic area | OATP1B1 | | OATP1B3 | | BCRP | |
| --- | --- | --- | --- | --- | --- | --- | --- |
|  |  | **N** | **%** | **N** | **%** | **N** | **%** |
| A | Alimentary tract and metabolism | 11 | 6.9 | 11 | 7.0 | 13 | 7.3 |
| B | Blood and blood forming organs | 7 | 4.4 | 6 | 3.8 | 9 | 5.1 |
| C | Cardiovascular system | 8 | 5.0 | 8 | 5.1 | 8 | 4.5 |
| D | Dermatological drugs | 0 | 0.0 | 0 | 0.0 | 1 | 0.6 |
| G | Genitourinary system and reproductive hormones | 2 | 1.3 | 2 | 1.3 | 3 | 1.7 |
| H | Systemic hormonal preparations | 3 | 1.9 | 3 | 1.9 | 3 | 1.7 |
| J | Antiinfectives for systemic use | 31 | 19.5 | 31 | 19.7 | 31 | 17.5 |
| L | Antineoplastic and immunomodulating agents | 69 | 43.4 | 67 | 42.7 | 81 | 45.8 |
| M | Musculoskeletal system | 3 | 1.9 | 4 | 2.5 | 2 | 1.1 |
| N | Nervous system | 16 | 10.1 | 16 | 10.2 | 18 | 10.2 |
| R | Respiratory system | 4 | 2.5 | 4 | 2.5 | 3 | 1.7 |
| V | Various ATC structures | 5 | 3.1 | 5 | 3.2 | 5 | 2.8 |
| Total |  | 159 |  | 157 |  | 177 |  |

**Table S3**. Number of approved products with text on OATP1B1, 1B3 or BCRP per approval year

|  | Number of products | | |
| --- | --- | --- | --- |
| Approval year | OATP1B1 | OATP1B3 | BCRP |
| 2012 | 6 | 6 | 8 |
| 2013 | 11 | 10 | 14 |
| 2014 | 12 | 13 | 13 |
| 2015 | 18 | 16 | 18 |
| 2016 | 12 | 12 | 11 |
| 2017 | 15 | 13 | 17 |
| 2018 | 7 | 8 | 13 |
| 2019 | 13 | 13 | 15 |
| 2020 | 21 | 22 | 19 |
| 2021 | 19 | 20 | 22 |
| 2022 | 19 | 18 | 21 |
| 2023* | 6 | 6 | 6 |
| Total 2012-2023 | 159 | 157 | 177 |

*Until September 7^th^

**Table S4** Type of data described in the SmPC text, categorised by clinical relevance and signal (all or positive only) for OATP1B1, 1B3 and BCRP

OATP1B1

|  | All | Clinical relevance yes | Clinical relevance unknown | Clinical relevance no |
| --- | --- | --- | --- | --- |
| In vitro data | 120 | 10 | 14 | 96 |
| In vivo data | 12 | 6 | 1 | 5 |
| Both | 27 | 10 | 1 | 16 |
| Sum | 159 | 26 | 16 | 117 |
|  |  |  |  |  |
| positive perpetrator signal only |  |  |  |  |
| In vitro data | 26 | 10 | 11 | 5 |
| In vivo data | 7 | 4 | 1 | 2 |
| Both | 12 | 6 | 1 | 5 |
| Sum | 45 | 20 | 13 | 12 |
|  |  |  |  |  |
| positive victim signal only |  |  |  |  |
| In vitro data | 6 | 0 | 3 | 3 |
| In vivo data | 2 | 1 | 0 | 1 |
| Both | 7 | 3 | 0 | 4 |
| Sum | 15 | 4 | 3 | 8 |

OATP1B3

|  | All | Clinical relevance yes | Clinical relevance unknown | Clinical relevance no |
| --- | --- | --- | --- | --- |
| In vitro data | 123 | 8 | 8 | 107 |
| In vivo data | 11 | 6 | 0 | 5 |
| Both | 23 | 8 | 1 | 14 |
| Sum | 157 | 22 | 9 | 126 |
|  |  |  |  |  |
| positive perpetrator signal only | | | | |
| In vitro data | 18 | 8 | 4 | 6 |
| In vivo data | 6 | 4 | 0 | 2 |
| Both | 11 | 6 | 1 | 4 |
| Sum | 35 | 18 | 5 | 12 |
|  |  |  |  |  |
| positive victim signal only | | | |  |
| In vitro data | 7 | 0 | 3 | 4 |
| In vivo data | 2 | 1 | 0 | 1 |
| Both | 5 | 2 | 0 | 3 |
| Sum | 14 | 3 | 3 | 8 |

BCRP

|  | All | Clinical relevance yes | Clinical relevance unknown | Clinical relevance no |
| --- | --- | --- | --- | --- |
| In vitro | 125 | 30 | 22 | 73 |
| In vivo | 16 | 7 | 1 | 8 |
| Both | 36 | 25 | 2 | 9 |
| SUM | 177 | 62 | 25 | 90 |
|  |  |  |  |  |
| positive perpetrator signal only | | | | |
| In vitro data | 46 | 26 | 13 | 7 |
| In vivo data | 8 | 5 | 1 | 2 |
| Both | 24 | 18 | 2 | 4 |
| SUM | 78 | 49 | 16 | 13 |
|  |  |  |  |  |
| positive victim signal only | | | | |
| In vitro data | 39 | 11 | 10 | 18 |
| In vivo data | 4 | 4 | 0 | 0 |
| Both | 22 | 15 | 1 | 6 |
| SUM | 65 | 30 | 11 | 24 |

Clinical relevance: whenever several interactions were described for a product, the classification reflects the highest clinical relevance (ie yes > unknown > no). For category “yes”, the text had to contain an actionable recommendation. Category “no” were those texts stipulating that there is no in vitro inhibition or that the API is not a substrate of the transporter, or those with in vivo results were the DDI was not considered clinically relevant. The category “unknown” covered texts explicitly stating that relevance was unknown and texts that could not be classified into the other categories.

**Table S5** SmPC text content (all sections) categorised into “perpetrator”, “victim” or “other” for OATP1B1, 1B3 and BCRP. The presence of an interaction (positive signal) is denoted with a plus sign, and the absence of an interaction (negative signals) is denoted with a minus sign. N/A stands for not available, meaning that the interaction text lacks information on that aspect (victim or perpetrator).

|  | OATP1B1 |  | OATP1B3 |  | BCRP |  |
| --- | --- | --- | --- | --- | --- | --- |
| Perpetrator/ victim | n | % | n | % | n | % |
| -/- -/NA and NA/- | 97 | 61 | 107 | 68 | 58 | 33 |
| +/+ | 5 | 3 | 4 | 3 | 30 | 17 |
| +/- | 16 | 10 | 10 | 6 | 17 | 10 |
| +/NA | 24 | 15 | 21 | 13 | 31 | 18 |
| -/+ | 6 | 4 | 6 | 4 | 23 | 13 |
| NA/+ | 4 | 3 | 4 | 3 | 12 | 7 |
| Other | 7 | 4 | 5 | 3 | 6 | 3 |
| SUM | 159 |  | 157 |  | 177 |  |

**Table S6**: Perpetrator text examples

| **Scenario** | **Perpetrator product (API)** | **SmPC text** |
| --- | --- | --- |
| Contraindication of concomitant use | N/A |  |
| Concomitant use not recommended or only with monitoring | Nubeqa (darolutamide) [7] | 4.4: Patients should be monitored for adverse reactions of BCRP, OATP1B1 and OATP1B3 substrates as co-administration with darolutamide may increase the plasma concentrations of these substrates. Darolutamide is an inhibitor of breast cancer resistance protein (BCRP) and Organic Anion Transporting Polypeptides (OATP) 1B1 and 1B3. Co-administration of rosuvastatin should be avoided unless there is no therapeutic alternative.  4.5: BCRP, OATP1B1 and OATP1B3 substrates  Darolutamide is an inhibitor of breast cancer resistance protein (BCRP) and Organic Anion Transporting Polypeptides (OATP) 1B1 and 1B3. Co-administration of rosuvastatin should be avoided unless there is no therapeutic alternative. Selection of an alternative concomitant medicinal product with less potential to inhibit BCRP, OATP1B1 and OATP1B3 should be considered. Administration of darolutamide (600 mg twice daily for 5 days) prior to co-administration of a single dose of rosuvastatin (5 mg) together with food resulted in approximately 5-fold increase in mean exposure (AUC) and Cmax of rosuvastatin. Co-administration of darolutamide with other BCRP substrates should be avoided where possible. Co-administration of darolutamide may increase the plasma concentrations of other concomitant BCRP, OATP1B1 and OATP1B3 substrates (e.g. methotrexate, sulfasalazine, fluvastatin, atorvastatin, pitavastatin). Therefore, it is recommended to monitor patients for adverse reactions of BCRP, OATP1B1 and OATP1B3 substrates. In addition, the related recommendation in the product information of these substrates should be followed when co-administered with darolutamide. |
| Monitoring recommended including dose adjustment | Rukobia (fostemsavir) [8] | 4.4: Dose modifications and/or careful titration of dose is recommended for certain statins that are substrates of OATP1B1/3 or BCRP (rosuvastatin, atorvastatin, pitavastatin, simvastatin and fluvastatin) when co-administered with fostemsavir (see section 4.5). […]  When fostemsavir is co-administered with tenofovir alafenamide (TAF), temsavir is expected to increase plasma concentrations of TAF via inhibition of OATP1B1/3 and/or BCRP. The recommended dose of TAF is 10 mg when co-administered with fostemsavir (see section 4.5).  4.5: In vitro, temsavir inhibited OATP1B1 and OATP1B3 (IC50 = 32 and 16 µM, respectively). Additionally, temsavir and its two metabolites (BMS-646915 and BMS-930644) inhibited BCRP (IC50 = 12, 35, and 3.5 to 6.3 µM, respectively). Based on these data, temsavir is expected to affect the pharmacokinetics of active substances that are substrates of OATP1B1/3 or BCRP (e.g. rosuvastatin, atorvastatin, simvastatin, pitavastatin and fluvastatin). Therefore, dose modifications and/or careful titration of dose is recommended for certain statins. [interaction table] |
| Concomitant use not recommended and dose staggering | Fotivda (tivozanib) [9] | 4.5: Tivozanib inhibits the transporter protein BCRP in vitro, but the clinical relevance of this finding is unknown (see section 5.2). Caution should be exercised if tivozanib is co-administered with rosuvastatin. Alternatively, a statin not subject to restriction of intestinal absorption by BCRP should be considered. Patients taking an oral BCRP substrate with a clinically-relevant efflux interaction in the gut should ensure that a suitable time window (e.g. 2 hours) is applied between administration of tivozanib and the BCRP substrate.  5.2: Tivozanib inhibits the transporter protein BCRP in vitro, at concentrations that are likely to restrict the effect to intestinal BCRP activity in vivo. |
|  | Jakavi (ruxolitinib) [10] | 4.5: Ruxolitinib may inhibit P-glycoprotein and breast cancer resistance protein (BCRP) in the intestine. This may result in increased sytemic exposure of substrates of these transporters, such as dabigatran etexilate, ciclosporin, rosuvastatin and potentially digoxin. Therapeutic drug monitoring (TDM) or clinical monitoring of the affected substance is advised. It is possible that the potential inhibition of P-gp and BCRP in the intestine can be minimised if the time between administrations is kept apart as long as possible.  5.2: In vitro data indicate that ruxolitinib may inhibit P-gp and BCRP. |
|  | Mekinist (trametinib) [11] | 4.5: Trametinib may result in transient inhibition of BCRP substrates (e.g. pitavastatin) in the gut, which may be minimised with staggered dosing (2 hours apart) of these agents and trametinib.  5.2: […] transient inhibition of BCRP substrates in the gut may occur (see section 4.5). |
|  | Imbruvica (ibrutinib)[12] | 4.5: Ibrutinib is a P-gp and breast cancer resistance protein (BCRP) inhibitor in vitro. As no clinical data are available on this interaction, it cannot be excluded that ibrutinib could inhibit intestinal P-gp and BCRP after a therapeutic dose. To minimise the potential for an interaction in the GI tract, oral narrow therapeutic range, P-gp or BCRP substrates such as digoxin or methotrexate should be taken at least 6 hours before or after IMBRUVICA. Ibrutinib may also inhibit BCRP in the liver and increase the exposure of medicinal products that undergo BCRP-mediated hepatic efflux, such as rosuvastatin.  5.2: Ibrutinib is an in vitro inhibitor of P-gp and BCRP (see section 4.5). |
| Precautions when no in vivo data are available | Rozlytrek (entrectinib) [13] | 4.5: Inhibition of BCRP was observed in in vitro studies. The clinical relevance of this inhibition is unknown, but caution is advised when sensitive oral BCRP substrates (e.g. methotrexate, mitoxantrone, topotecan, lapatinib) are co-administered with entrectinib, due to the risk of increased absorption. […] In vitro data indicate that entrectinib has weak inhibitory potential towards organic anion-transporting polypeptide (OATP)1B1. The clinical relevance of this inhibition is unknown, but caution is advised when sensitive oral OATP1B1 substrates (e.g. atorvastatin, pravastatin, rosuvastatin repaglinide, bosentan) are co-administered with entrectinib, due to the risk of increased absorption. |
|  | Dovprela (pretomanid) [14] | 4.5: In vitro studies indicate that pretomanid is an inhibitor of BCRP, OATP1B3 and P-gp. No clinical studies have been performed to investigate these interactions. Therefore, it cannot be excluded that co-administration of pretomanid with sensitive OATP1B3 substrates (e.g., valsartan, statins), BCRP substrates (e.g. rosuvastatin, prazosin, glyburide, sulfasalazine) and P-gp substrates (e.g. digoxin, dabigatran etexilate, verapamil) may increase their exposure. If pretomanid is co-administered with substrates of OATP1B3, BCRP or P-gp, monitoring for drug-related adverse reactions to the coadministered medicinal product should be performed.  5.2: The potential of pretomanid to inhibit P gp, OATP1B3, OCT2 and BCRP has not been investigated at clinically relevant concentrations. |
| In vitro data with no clinical relevance | Iclusig (ponatinib) [15] | 5.2 At therapeutic serum concentrations, ponatinib did not inhibit OATP1B1 or OATP1B3 […]. |

**Table S7**: Victim text examples

| **Scenario** | **Victim product (API)** | **SmPC text** |
| --- | --- | --- |
| Contraindication of concomitant use | Zepatier (elbasvir, gazoprevir) [16] | 4.3: Co-administration with inhibitors of organic anion transporting polypeptide 1B (OATP1B), such as rifampicin, atazanavir, darunavir, lopinavir, saquinavir, tipranavir, cobicistat or ciclosporin.  4.4: Co-administration of ZEPATIER and OATP1B inhibitors is contraindicated because it may significantly increase grazoprevir plasma concentrations.  4.5: Grazoprevir is a substrate of OATP1B drug transporters. Co-administration of ZEPATIER with medicinal products that inhibit OATP1B transporters is contraindicated because it may result in a significant increase in the plasma concentration of grazoprevir. |
| Concomitant use not recommended or only with monitoring | Venclyxto (venetoclax) [17] | 4.4: [in a warning for tumor lysis syndrome] Also, inhibitors of P-gp or BCRP may increase venetoclax exposure (see section 4.5).  4.5: Co-administration of 400 mg once daily ketoconazole, a strong CYP3A, P-gp and BCRP inhibitor, for 7 days in 11 patients increased venetoclax Cmax to 2.3-fold and AUC to 6.4-fold. […]  Venetoclax is a substrate for P-gp and BCRP. Co-administration of a 600 mg single dose of rifampicin, a P-gp inhibitor, in 11 healthy subjects increased venetoclax Cmax by 106% and AUC by 78%. Concomitant use of venetoclax with P-gp and BCRP inhibitors at initiation and during the dose-titration phase should be avoided; if a P-gp and BCRP inhibitor must be used, patients should be monitored closely for signs of toxicities (see section 4.4). |
| Monitoring recommended including dose adjustment | Aquipta (atogepant) [18] | 4.2: Dosing modifications for concomitant use of specific medicinal products are provided in Table 1. Strong OATP inhibitors: 10 mg.  4.5: Organic anion transporting polypeptide (OATP) inhibitors (e.g., rifampicin, ciclosporin, ritonavir) can significantly increase systemic exposure to atogepant. Co-administration of atogepant with single dose rifampicin resulted in increased exposure (Cmax by 2.23-fold and AUC by 2.85-fold) of atogepant in healthy subjects (see section 4.2).  5.2: Atogepant is a substrate of P-gp, BCRP, OATP1B1, OATP1B3, and OAT1. Dose adjustment for concomitant use with strong inhibitors of OATP is recommended based on a clinical interaction study with a strong OATP inhibitor. |
|  | Adempas (riociguat) [19] | 4.2: Patients on stable doses of strong multi pathway CYP / P-glycoprotein (P-gp) and breast cancer resistance protein (BCRP) inhibitors  Coadministration of riociguat with strong multi pathway CYP and P-gp/BCRP inhibitors such as azole antimycotics (e.g. ketoconazole, itraconazole) or HIV protease inhibitors (e.g. ritonavir) increases exposure to riociguat (see section 4.5). When initiating riociguat in patients on stable doses of strong multi pathway CYP and P-gp/BCRP inhibitors, consider a starting dose of 0.5 mg 3 times a day to mitigate the risk of hypotension. Monitor for signs and symptoms of hypotension on initiation and on treatment. Consider a dose reduction for patients on riociguat doses higher than or equal to 1.0 mg if the patient develops signs or symptoms of hypotension (see sections 4.4 and 4.5). No clinical data is available in children receiving concomitant systemic treatment with strong CYP/Pgp and BCRP inhibitors.  4.4: The concomitant use of riociguat with strong multi pathway CYP and P-gp / BCRP inhibitors such as azole antimycotics (e.g. ketoconazole, posaconazole, itraconazole) or HIV protease inhibitors (e.g. ritonavir) results in a pronounced increase in riociguat exposure (see sections 4.5 and 5.2). Assess the benefit-risk for each patient individually before prescribing riociguat in patients on stable doses of strong multi pathway CYP and P-gp/BCRP inhibitors. To mitigate the risk of hypotension, consider dose reduction and monitoring for signs and symptoms of hypotension(see sections 4.2 and 4.5).In patients on stable doses of riociguat, the initiation of strong multi pathway CYP and P-gp/BCRP inhibitors is not recommended as no dosage recommendation can be given due to limited data. Alternative treatments should be considered. The concomitant use of riociguat with strong CYP1A1 inhibitors, such as the tyrosine kinase inhibitor erlotinib, and strong P-glycoprotein (P-gp) / breast cancer resistance protein (BCRP) inhibitors, such as the immuno-suppressive agent cyclosporine A, may increase riociguat exposure (see sections 4.5 and 5.2). These medicinal products should be used with caution. Blood pressure should be monitored and dose reduction of riociguat be considered.  4.5: To mitigate the risk of hypotension when riociguat is initiated in patients on stable doses of strong multi pathway CYP (especially CYP1A1 and CYP3A4) and P-gp/BCRP inhibitors, e.g. as contained in HAART, consider a reduced starting dose. It is recommended to monitor these patients for signs and symptoms of hypotension (see sections 4.2 and 4.4).  Antifungals  In vitro, ketoconazole, classified as a strong CYP3A4 and P-glycoprotein (P-gp) inhibitor, has been  shown to be a multi-pathway CYP and P-gp/breast cancer resistance protein (BCRP) inhibitor for riociguat metabolism and excretion (see section 5.2). Concomitant administration of 400 mg once daily ketoconazole led to a 150% (range up to 370%) increase in riociguat mean AUC and a 46% increase in mean Cmax. Terminal half-life increased from 7.3 to 9.2 hours and total body clearance decreased from 6.1 to 2.4 L/h. To mitigate the risk of hypotension when riociguat is initiated in patients on stable doses of strong multi pathway CYP (especially CYP1A1 and CYP3A4) and P-gp/BCRP inhibitors, e.g. ketoconazole, posaconazole or itraconazole consider a reduced starting dose. It is recommended to monitor these patients for signs and symptoms of hypotension (see sections 4.2 and 4.4).  Concomitant use with other CYP and P-gp/BCRP inhibitors  Medicinal products strongly inhibiting P-gp/BCRP such as the immuno-suppressive cyclosporine A, should be used with caution (see sections 4.4 and 5.2).  5.2: Based on in vitro data riociguat and its main metabolite are substrates of the transporter proteins P-gp (P-glycoprotein) and BCRP (breast cancer resistance protein). |
| Concomitant use not recommended and dose staggering | Giotrif (afatinib) [20] | 4.5 In vitro studies have demonstrated that afatinib is a substrate of P-gp and BCRP. When the strong P-gp and BCRP inhibitor ritonavir (200 mg twice a day for 3 days) was administered 1 hour before a single dose of 20 mg GIOTRIF, exposure to afatinib increased by 48% (area under the curve (AUC0-∞)) and 39% (maximum plasma concentration (Cmax)). In contrast, when ritonavir was administered simultaneously or 6 hours after 40 mg GIOTRIF, the relative bioavailability of afatinib was 119% (AUC0-∞) and 104% (Cmax) and 111% (AUC0-∞) and 105% (Cmax), respectively. Therefore, it is recommended to administer strong P-gp inhibitors (including but not limited to ritonavir, cyclosporine A, ketoconazole, itraconazole, erythromycin, verapamil, quinidine, tacrolimus, 7 nelfinavir, saquinavir, and amiodarone) using staggered dosing, preferably 6 hours or 12 hours apart from GIOTRIF. |
| Precautions when no in vivo data are available | Talzenna (talazoparib) [21] | 4.5: Talazoparib is a substrate for drug transporters P-gp and Breast Cancer Resistance Protein (BCRP) and it is mainly eliminated by renal clearance as unchanged compound. […] The effect of BCRP inhibitors on PK of talazoparib has not been studied in vivo. Co-administration of talazoparib with BCRP inhibitors may increase talazoparib exposure. Concomitant use of strong BCRP inhibitors (including but not limited to curcumin and cyclosporine) should be avoided. If co-administration of strong BCRP inhibitors cannot be avoided, patient should be monitored for potential increased adverse reactions.  5.2: Talazoparib is a substrate of P-gp and BCRP transporters. |
|  | Rubraca (rucaparib) [22] | 4.5: In vitro, rucaparib was shown to be a substrate of P-gp and BCRP. Effect of P-gp and BCRP inhibitors on rucaparib PK cannot be ruled out. Caution is recommended when rucaparib is co-administered with medicinal products that are strong inhibitors of P-gp.  5.2: In vitro, rucaparib was shown to be a substrate of P-gp and BCRP, but not a substrate of renal uptake transporters OAT1, OAT3, and OCT2, or hepatic transporters OAPT1B1 and OATP1B3. Effect of Pgp and BCRP inhibitors on rucaparib PK cannot be ruled out. |
|  | Stivarga (regorafenib)[23] | 4.5: In vitro studies indicate that the active metabolites M-2 and M-5 are substrates for P-glycoprotein and BCRP. Inhibitors and inducers of BCRP and P-glycoprotein may interfere with the exposure of M-2 and M-5. The clinical significance of these findings is unknown (see also section 5.2). 5.2: Metabolite M-5 is a weak BCRP-substrate. |
|  | Mavenclad (cladribine) [24] | 4.5: At the level of cladribine absorption, the only conceivable interaction pathway of clinical relevance appears to be the breast cancer resistance protein (BCRP or ABCG2). Inhibition of BCRP in the gastrointestinal tract may increase the oral bioavailability and systemic exposure of cladribine. Known BCRP inhibitors, which may alter the pharmacokinetics of BCRP substrates by 20% in vivo, include eltrombopag. […] Although the clinical relevance of such interactions is unknown, it is recommended that coadministration of potent ENT1, CNT3 or BCRP inhibitors be avoided during the4- to 5-day cladribine treatment. If this is not possible, selection of alternative concomitant medicinal products with no, or minimal ENT1, CNT3 or BCRP transporter inhibiting properties should be considered. If this is not possible, dose reduction to the minimum mandatory dose of medicinal products containing these compounds, separation in the timing of administration and careful patient monitoring is recommended. […]The effects of potent inducers of the efflux transporters BCRP and P-glycoprotein (P-gp) on the bioavailability and disposition of cladribine have not been formally studied. A possible decrease in cladribine exposure should be considered if potent BCRP (e.g. corticosteroids) or P-gp (e.g. rifampicin, St. John's Wort) transporter inducers are co-administered. 5.2: The distribution of cladribine across biological membranes is facilitated by various transport proteins, including ENT1, CNT3 and BCRP. |
| In vitro data with no clinical relevance | Iclusig (ponatinib) [15] | 5.2 Ponatinib is not a substrate for the human organic anion transporting polypeptides OATP1B1, OATP1B3 and the organic cation transporter OCT-1. |

**References**

1.European Medicines Agency (2015) Ofev (nintedanib) SmPC <https://www.ema.europa.eu/en/documents/product-information/ofev-epar-product-information_en.pdf>. Accessed 2023-09-07

2.European Medicines Agency (2014) Vargatef (nintedanib) SmPC <https://www.ema.europa.eu/en/documents/product-information/vargatef-epar-product-information_en.pdf>. Accessed 2023-09-07

3.European Medicines Agency (2017) Vemlidy (tenofovir alafenamide) SmPC <https://www.ema.europa.eu/en/documents/product-information/vemlidy-epar-product-information_en.pdf>. Accessed 2023-09-07

4.European Medicines Agency (2019) Viekirax (ombitasvir, paritaprevir and ritonavir) SmPC <https://www.ema.europa.eu/en/documents/product-information/viekirax-epar-product-information_en.pdf>. Accessed 2023-09-07

5.European Medicines Agency (2006) Norvir (ritonavir) SmPC <https://www.ema.europa.eu/en/documents/product-information/norvir-epar-product-information_en.pdf>. Accessed 2023-09-07

6.European Medicines Agency (2024) ICH M12 Guideline on drug interaction studies, ICH M12 Step 5 <https://www.ema.europa.eu/en/documents/scientific-guideline/ich-m12-guideline-drug-interaction-studies-step-5_en.pdf>

7.European Medicines Agency (2020) Nubeqa (darolutamide) SmPC <https://www.ema.europa.eu/en/documents/product-information/nubeqa-epar-product-information_en.pdf>. Accessed 2023-09-07

8.European Medicines Agency (2021) Rukobia (fostemsavir) SmPC <https://www.ema.europa.eu/en/documents/product-information/rukobia-epar-product-information_en.pdf>. Accessed 2023-09-07

9.European Medicines Agency (2022) Fotivda (tivozanib) SmPC <https://www.ema.europa.eu/en/documents/product-information/fotivda-epar-product-information_en.pdf>. Accessed 2023-09-07

10.European Medicines Agency (2017) Jakavi (ruxolitinib) SmPC <https://www.ema.europa.eu/en/documents/product-information/jakavi-epar-product-information_en.pdf>. Accessed 2023-09-07

11.European Medicines Agency (2019) Mekinist (trametinib) SmPC <https://www.ema.europa.eu/en/documents/product-information/mekinist-epar-product-information_en.pdf>. Accessed 2023-09-07

12.European Medicines Agency (2019) Imbruvica (ibrutinib) SmPC <https://www.ema.europa.eu/en/documents/product-information/imbruvica-epar-product-information_en.pdf>. Accessed 2023-09-07

13.European Medicines Agency (2022) Rozlytrek (entrectinib) SmPC <https://www.ema.europa.eu/en/documents/product-information/rozlytrek-epar-product-information_en.pdf>. Accessed 2023-09-07

14.European Medicines Agency (2023) Dovprela (pretomanid) SmPC <https://www.ema.europa.eu/en/documents/product-information/dovprela-previously-pretomanid-fgk-epar-product-information_en.pdf>. Accessed 2023-09-07

15.European Medicines Agency (2018) Iclusig (ponatinib) SmPC <https://www.ema.europa.eu/en/documents/product-information/iclusig-epar-product-information_en.pdf>. Accessed 2023-09-07

16.European Medicines Agency (2021) Zepatier (elbasvir, grazoprevir) SmPC <https://www.ema.europa.eu/en/documents/product-information/zepatier-epar-product-information_en.pdf>. Accessed 2023-09-07

17.European Medicines Agency (2018) Venclyxto (venetoclax) SmPC <https://www.ema.europa.eu/en/documents/product-information/venclyxto-epar-product-information_en.pdf>. Accessed 2023-09-07

18.European Medicines Agency (2023) Aquipta (atogepant) SmPC <https://www.ema.europa.eu/en/documents/product-information/aquipta-epar-product-information_en.pdf>. Accessed 2023-09-07

19.European Medicines Agency (2019) Adempas (riociguat) SmPC <https://www.ema.europa.eu/en/documents/product-information/adempas-epar-product-information_en.pdf>. Accessed 2023-09-07

20.European Medicines Agency (2018) Giotrif (afatinib) SmPC <https://www.ema.europa.eu/en/documents/product-information/giotrif-epar-product-information_en.pdf>. Accessed 2023-09-07

21.European Medicines Agency (2019) Talzenna (talazoparib) SmPC <https://www.ema.europa.eu/en/documents/product-information/talzenna-epar-product-information_en.pdf>. Accessed 2023-09-07

22.European Medicines Agency (2022) Rubraca (rucaparib) SmPC <https://www.ema.europa.eu/en/documents/product-information/rubraca-epar-product-information_en.pdf>. Accessed 2023-09-07

23.European Medicines Agency (2018) Stivarga (regorafenib) SmPC <https://www.ema.europa.eu/en/documents/product-information/stivarga-epar-product-information_en.pdf>. Accessed 2023-09-07

24.European Medicines Agency (2022) Mavenclad (cladribine) SmPC <https://www.ema.europa.eu/en/documents/product-information/mavenclad-epar-product-information_en.pdf>. Accessed 2023-09-07
